# Unmanned Aerial Vehicle for Laser Based Biomedical Sensor Development and Examination of Device Trajectory

**DOI:** 10.3390/s22093413

**Published:** 2022-04-29

**Authors:** Usman Masud, Tareq Saeed, Faraz Akram, Hunida Malaikah, Altaf Akbar

**Affiliations:** 1 Faculty of Electrical and Electronics Engineering, University of Engineering and Technology, Taxila 47050, Pakistan; 2 Department of Electrical Communication Engineering, University of Kassel, 34127 Kassel, Germany; 3 Nonlinear Analysis and Applied Mathematics (NAAM)-Research Group, Department of Mathematics, Faculty of Science, King Abdulaziz University, P.O. Box 80203, Jeddah 21589, Saudi Arabia; tsalmalki@kau.edu.sa; 4 Faculty of Engineering and Applied Sciences, Riphah International University, Islamabad 44000, Pakistan; faraz.akram@riphah.edu.pk; 5 Department of Mathematics, Faculty of Science, King Abdulaziz University, P.O. Box 80203, Jeddah 21589, Saudi Arabia; hmalaikah@kau.edu.sa; 6 Department of Economics, Management, Industrial Engineering and Tourism (DEGEIT), University of Aveiro, 3800-000 Aveiro, Portugal

**Keywords:** unmanned aerial vehicle, spectroscopy, brain–computer interface application, mathematical modelling, semiconductor laser

## Abstract

Controller design and signal processing for the control of air-vehicles have gained extreme importance while interacting with humans to form a brain–computer interface. This is because fewer commands need to be mapped into multiple controls. For our anticipated biomedical sensor for breath analysis, it is mandatory to provide medication to the patients on an urgent basis. To address this increasingly tense situation in terms of emergencies, we plan to design an unmanned vehicle that can aid spontaneously to monitor the person’s health, and help the physician spontaneously during the rescue mission. Simultaneously, that must be done in such a computationally efficient algorithm that the minimum amount of energy resources are consumed. For this purpose, we resort to an unmanned logistic air-vehicle which flies from the medical centre to the affected person. After obtaining restricted permission from the regional administration, numerous challenges are identified for this design. The device is able to lift a weight of 2 kg successfully which is required for most emergency medications, while choosing the smallest distance to the destination with the GPS. By recording the movement of the vehicle in numerous directions, the results deviate to a maximum of 2% from theoretical investigations. In this way, our biomedical sensor provides critical information to the physician, who is able to provide medication to the patient urgently. On account of reasonable supply of medicines to the destination in terms of weight and time, this experimentation has been rendered satisfactory by the relevant physicians in the vicinity.

## 1. Introduction

Over the past decades, computer and communication technologies have developed rapidly in the form of brain–computer interface (BCI). As evident from its underlying nomenclature, BCI enables a user to control a computer or any other device with signals of the brain. In the past couple of decades, researchers have developed several applications of the BCI including, but not limited to, character spelling [1,2] and word typing [3,4], wheelchair control [5,6], prosthetics control [7,8], neurological rehabilitation [9], home control [10], virtual reality control [11], gaming [12,13] and quadcopter control [14,15]. Taking these into account, this paper aims at developing an Unmanned Aerial Vehicle (UAV) for the BCI application for better operating of air-vehicles.

Therefore, these techniques have now become an indispensable tool for patients’ daily life. The purpose of BCI is to make patients’ life more appropriate and natural in a daily living environment [16]. The fundamental aim of BCI is to assist patients (particularly, in locked-in state) to interact with the living environment using only brain signals [17,18]. After the obtained command from the brain, another challenge is to properly control the applications that manipulate wheelchairs, robotic arms or drones [19,20,21,22,23,24,25] as in this study. Many signal processing techniques have previously been created, however, in this study, a framework is devised that can be helpful in finer operation of air-vehicles. Several techniques can be implemented for this purpose, as in [26,27].

Biomedical logistic sensors are gaining interest in the aerospace industry, extending their applications from solar-powered drones to origami-style space-based solar power stations due to their flexibility, light weight and transparency. The basic element is an autonomous air-vehicle which is a quad-rotor without the presence of a human pilot onboard [28,29,30]. Its navigation or movement is controlled through a control system on board or it can also be navigated manually by remote control from the ground [31,32,33,34]. A quad-rotor has three translational and rotational movements through which it can achieve six degrees of freedom. For this purpose, the rotational and translational motion have to couple with the help of rotors [35,36]. The quad-copter has four arms and each arm contains an independent rotor. Generally, quad-rotors use two pairs of propellers with identical parameters (i.e., two clockwise and two anti-clockwise). The variation in speed of each rotor makes it possible to achieve the manoeuvring of the quad-rotor.

The resulting dynamics of the model are highly nonlinear, especially after accounting for the complicated aerodynamics effects and unlike ground vehicles which have much friction during their motion, the quad-rotors may have little friction to gain their movement (as per their system model or design), and they must provide their own damping effects to eliminate all these nonlinear factors. For this purpose, these vehicles use an electronic control system to maintain the stability of quad-rotors using electronic board and sensors, i.e., an accelerometer [37,38,39,40,41,42,43]. The quad-rotors were among the first vehicles to take off and land vertically. The agile and revolutionary design of quad-rotors not only makes them capable of exploring an unknown locus, but they can also move with precision and much faster pace than any other vehicle in a dense environment [44,45]. This paper is a demonstration of one of the versatile applications of quad-rotors to transfer product autonomously to the required destination.

**Figure 1:**
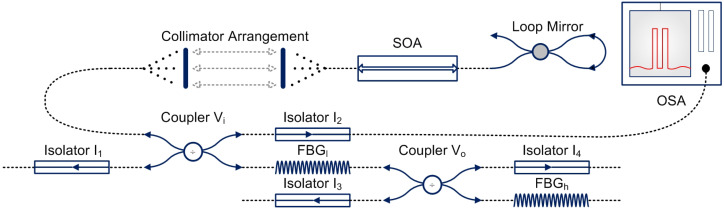
Design of the laser based two mode experimental scheme [38].

With this background, the main goal of our work is to develop a microsensor which is based on the fundamentals of two modes (wavelengths), utilising the principles of intracavity absorption spectroscopy [38,44]. This is a laser-based setup that consists of two wavelengths (specified by the term *modes*), as shown in Figure 1. The scheme works in a way in which the light from the Semiconductor Optical Amplifier (SOA) is reflected by two Fibre Bragg Gratings (FBGs), each specifying a wavelength (mode) that creates a competition between both modes. The difference between the intensities of both modes (SOA and human breath) aids in the detection of diseases of elderly patients, monitored continuously by the respective physician. The Variable Couplers (VCs) divide the light intensity, and the isolators ensure its flow in one direction (for instance, to the Optical Spectrum Analyzer (OSA)) (complete experimental details can be found in [46,47], with latest developments regarding expansion to wireless channel in [48]).

In case of any emergency situation, it is obvious from the description provided above that this demands spontaneous action by the physician in the form of transportation of medication to the said patient [39,44,45,49]. In light of this fact, we plan to make a product that is capable of transferring products, in particular, medical items from one place to another autonomously, with a limited time span. The physicians dealing with emergency situations were consulted for this purpose, and the foremost requirements were discussed. According to them, the said device must have reliable accuracy and efficiency that is mandatory in transportation procedure. As illustrated above, in case of an emergency, the patient must be immediately provided with aid in such a way that the life threatening situation can be avoided. This can be some suitable medicine or some medical instruments that can help as a life saver. This, in turn, means that the hardware and software parts must both be developed in a strictly technical way that helps in the operation of the device afterwards [40,42,43,50].

The proposed sensor will be used for the detection of Volatile Organic Compunds (VOCs) in the human breath which is exhaled from the lungs. This can provide useful information about specific diseases, hence the motivation behind this work. Certain sensors exist for the checking of specific health characteristics in human beings [51,52]. For instance, wearable sensors are being specifically developed that help to check the health of human beings on a continuous basis [51]. Molecularly imprinted materials are being widely used to sense human attributes [53]. Recent advancements include, but are not limited to, behaviour verification, movement monitoring, surveillance, characterization and other applications for human beings and animals [54,55]. On account of these notions, there exists a wide range of work for future in this specific area of development, as predicted in [56]. Hence, this is the first attempt that targets human breath and its diagnosis [38,55,57]. The list of abbreviations is outlined in Table 1.

*Motivation and objectives*

- BCIs have been successfully incorporated into numerous areas of the world, with successful outcomes;
- Biomedical sensors have been evolving fast for the past few decades and BCIs are proving to be a very important tool for that;
- During the state of an emergency for elderly patients, it is mandatory to attend and provide medication at the earliest to the relevant person, for which UAVs can be utilized effectively;
- Considering the development of biomedical sensor for the targeted patients, a UAV is planned to be designed that can aid in the remote monitoring and first aid to the said individuals;
- The quadcopter designed in this work has presented a highly stable operation, which is mandatory for the supply of medical equipment from the hospital to the patient.

## 2. Related Work

In the last few years, the relevance and applications of unmanned air-vehicles have multiplied significantly in different areas of life ([30,32,36,45,58,59]), based on various operational mechanisms. There are many advantages to these devices, including, but not limited to, the cheap potential use of these vehicles, which gives opportunities to perform tasks which are very difficult and sometimes impossible otherwise, especially for emergency operations [44,60]. Most of these vehicles are not only able to fly in complex and busy places, but also can reach them in much less time than people themselves, which is desirable in the modern era. Thanks to these merits, many research organizations in collaboration with vendors have started delivering products through multi-rotors after recognizing the capabilities of these flying vehicles [61,62,63]. However, being a recent area of investigation, these devices lack stability and appropriate designs for specific applications, which are the foremost disadvantages of using them. Another problem is the usage of expensive equipment that cannot be purchased for normal air-vehicles. For these reasons, the current focus is on the design and modelling of quad-rotors in order to make them stable, reliable and cheap. Frame design and modelling are the first steps toward the journey of building any air-vehicle, as the overall flight dynamics and parameters are based on its frame. It is therefore important to mention that the design of a quad-copter’s dynamics is classified with respect to two reference systems, i.e., body frame and inertial frame.

After discussions with the physicians working in the emergency, it is very important to realize that the device is designed for the transportation of medical items. For this purpose, there are several factors that must be considered beforehand. These include, but should not be limited to, efficient utilization of resources, supplying driving energy to the device in the form of its voltage, spectrum allocation and obtaining permission from the telecommunication authorities. Another prominent factor would be that the medical aspects have to be checked and examined to the maximum possible level of emergency items. To the best of our knowledge, this is the first approach in our vicinity in this connection, with limited financial resources and overall high efficiency, as discussed in the forthcoming sections in detail. The cases have been discussed in appropriate details in [64,65,66,67]. The trajectory of the quad-copter must be controlled in a reasonable way to do this, which involves its propulsion [68,69]. Propulsion means to push forward or to drive an object. In terms of an air-vehicle, propulsion is the force through which propellers push the air down and gain thrust. Brushless motors are mostly used to achieve propulsion in a quad-copter. These motors not only have very high power-to-weight ratio, but can also spin with thousands of revolutions per minute (rpm) [63]. The motor’s speed is controlled through an electronic device called an electronic speed controller (ESC) [70,71]. The ESC can switch motors from on to off state and vice versa, thereby maintaining the desired speed and thrust of quad-copter. Therefore, controlling the air-vehicle’s flight dynamics is a complex and interesting problem. The core unit of an air-vehicle is its control mechanism, which works like a brain in humans, and therefore has been the focus of interest in the last few years by the scientists via different approaches. Some model the control system directly by calculating their required parameters from the system and some use different theories to achieve these tasks (i.e., through classical control theory phenomenon) [36,37,58,62,64].

## 3. Hardware Considerations

Since the device is aimed for a biomedical application, there are numerous aspects that have to be brought into attention beforehand.

### 3.1. FRAME

The standout amongst the most vital piece of any quad-copter is its frame. It ought to be lightweight and flexible with the goal that it can hold the weight of different segments [72,73]. This is essential because the medical equipment can be of various weights. Moreover, the object to be moved might be sensitive in the sense that a slight damage during the operation could cause serious consequences [64]. Therefore, the option of constructing an edge at home, with the assistance of aluminium or balsa sheet, is eradicated. We resort to using a pre-fabricated casing whose parts are moderately low-cost and simple to supplant. In case of a crash, the arms ought to be somewhat resistant in a way that they should be the ones which undergo damage, thereby averting any harm to engines or costly gadgets on the edge. On the other hand, we really need them to be somewhat fragile, to accommodate the object on quad-copter.

Likewise, the arms assume an essential part in the battle against vibrations, which can cause various diverse issues. Flight controllers, with their touchy accelerometers and spinners, do not normally respond well to unremitting shaking. Any vibrations during the flight in a non-friendly environment may cause damage [74].

If we use an arm which has too much flexibility, it can reverberate and create harmonics that are transferred across the multi-rotor. On the contrary, arms that are too stiff would pass on vibrations with no hosing, bringing similar issues. There is a fine line between these two matters which needs to be drawn, and we attempt to use carbon fibre which is one of the most widely recognized materials for multi-rotor outlines. A large number of its physical properties suit our application [44,64].

### 3.2. Selection of Brush-Less DC (BLDC) Motor

BLDC (brush-less DC) motors do not utilise brushes for compensation. They are electronically commutated and have the following properties [72,75]:

- High effectiveness with noiseless tasks;
- Better speed versus torque attributes; and
- Quite high speed and longer life.

To control the speed of the motor, an ESC is required. The life of BLDC motor is quite long as there are no brushes which might be a source of damage. There is no starting and considerably less electrical commotion. In addition, considering the device for a biomedical application, the advantage of a brushless motor is its better preliminary cost ([44,65]).

We require four numbers of BLDC Motors for the copter. For the major part of the device, brushless motors are stated in kVs, such as, 810 kV, 1100 kV, 1400 kV, and 1800 kV. At this point, it must be recalled that the kV rating indicates the number of revolutions per minute (rpm) of the motor. For instance, a BLDC motor which has a kV rating of 1000 kV will turn at 1000 rpm when a voltage of 1 volt is applied at its input. Similarly, on applying 12 volts, the motor will turn at 12,000 rpm, and so on [63,75].

### 3.3. Electronic Speed Controller (ESC)

It is important to control the mechanism of the BLDC in a reliable way, for which an ESC is used. An ESC in a quad-copter performs two essential functions ([69,70]). First, it works as a Battery Elimination Circuit (BEC), a device that enables both the motors and the recipient to be powered by a solitary battery. The second function is to take the receiver’s feedback, in the form of the flight controller’s signal, and provide the required amount of current to the motors.

Each motor requires an ESC that regulates the amount of power to the engine, as indicated by the input throttle level. It additionally gives +5 V power to the flight hardware. ESC is based on a 32-bit microcontroller (ARM/AVR) and it has a variety of MOSFETs to drive the BLDC engine [63]. The firmware of ESC can be customized during the manufacturing phase.

In this way, the ESCs perform and ideal job of controlling BLDC. In simple words, an ESC is just a brushless motor controller board with battery input and a three-phase yield for the motor. ESCs can be found in a wide range of variations, where the input current is the most critical factor. This enables an appropriate control of the cut-off voltage, timing, acceleration and braking mechanism of the system [63,71,75].

### 3.4. Power Input

Lithium Polymer (Li-Po) battery is a kind of rechargeable battery that has overwhelmed the electric world, particularly for quad-copters. They are the primary reason why electric flight is presently an exceptionally feasible choice over fuel-controlled models.

Li-Po batteries are light in weight and hold enormous power in a little bundle. However, they are costly and have a lifetime of just 400 to 500 charge cycles. This means that a lot of care has to be to be taken while using this device. Otherwise, due to the unpredictable electrolyte utilized as a part of Li-Po batteries, they can blast or burst into flames easily when misused. Every cell of the Li-Po battery is rated at 3.7 V. The Li-Po battery, with four cells each of 3.7 V, used for our project is rated at 14.8 V. The amount of power the battery pack can hold is called the limit, and it is shown in milliamp hours (mAh). The battery we use for our project is of 3000 mAh. This means that 3000 mAh would be totally released in one hour when a 3000 mA stack is placed on it.

### 3.5. Propellers

It is imperative to rotate the motor, and hence the device. Therefore, on each of the brushless motors, a propeller is mounted. The four propellers are really not indistinguishable. When one takes a photo from below, one sees that the front and the back propellers are tilted to one side, while the left and right propellers are tilted to one side.

## 4. Design Methodology

Here, the procedure we choose to accomplish our project is discussed with the help of a spiral model methodology ([76,77]). We include mathematical approaches and algorithms that are implemented throughout in this project which helps in better explaining the kinematics and aerodynamics of the quad-copter.

### 4.1. Flowchart

**Figure 2:**
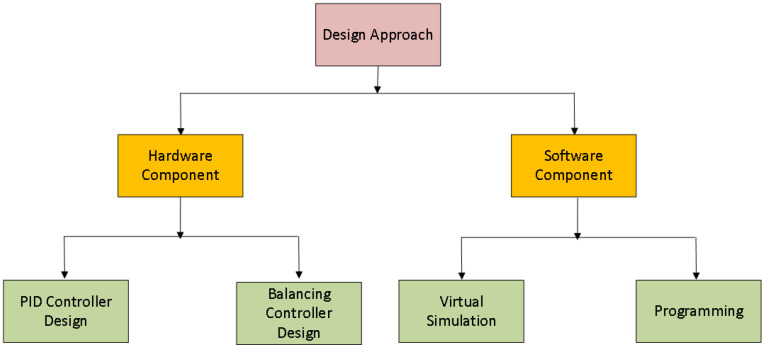
Design Methodology.

**Figure 3:**
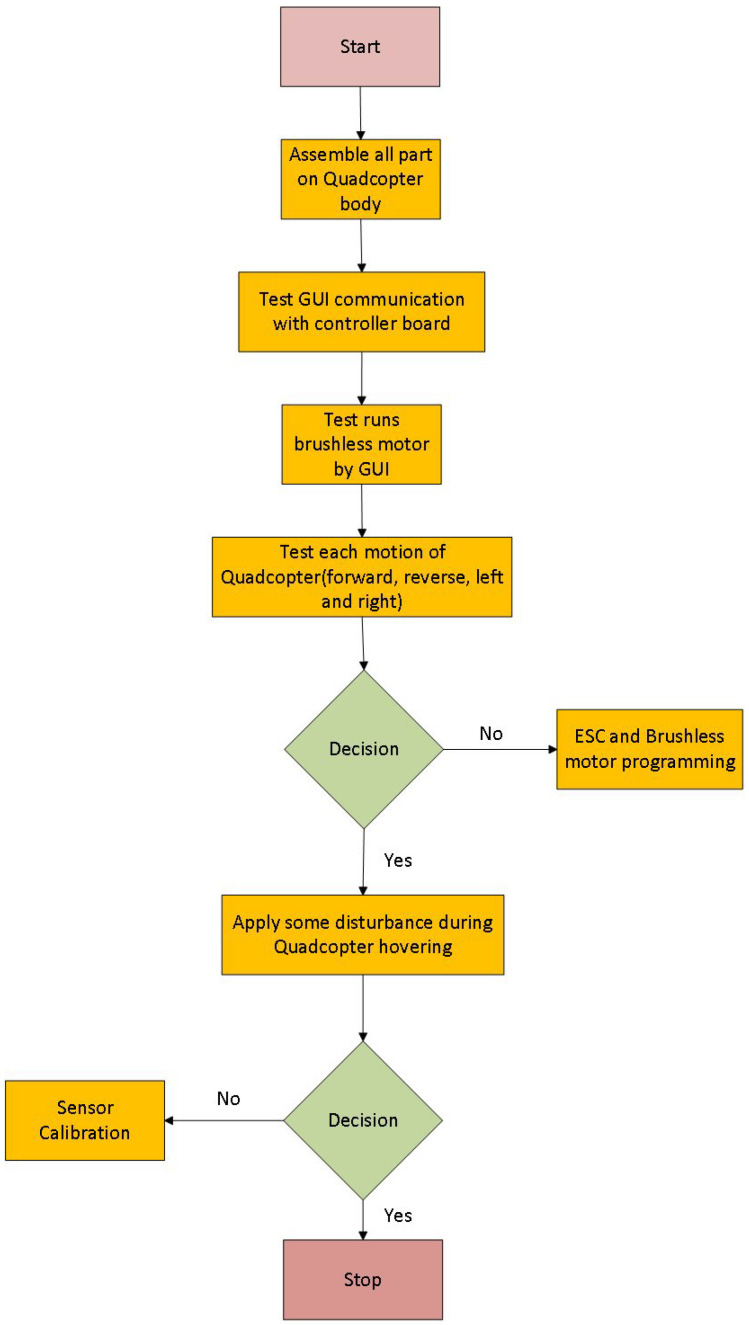
Flow chart of assembling and testing quad-copter.

The design of the quad-copter is divided into two stages. In the first stage, hardware design and assembling of quad-copter is performed, while in the second stage, software design and its implementation is completed, as shown in Figure 2. For a better explanation, the flowchart of quad-copter design is also shown in the Figure 3.

With the help of the equipment mentioned above, we proceed in the following way. The components are assembled to establish a physical structure of the quad copter, followed by testing the GUI communication with the controller board. Test runs are conducted on the brushless motors by this GUI, checking its forward, reverse, left and right movements, respectively. Afterwards, the ESC and brushless motors are programmed in case there is some issue with the said movement. This is cross-checked by applying some disturbance to the quad copter, and the sensor is calibrated to accommodate the said level of disturbance.

### 4.2. Mathematical Modelling of Quad-Copter Dynamics

**Figure 4:**
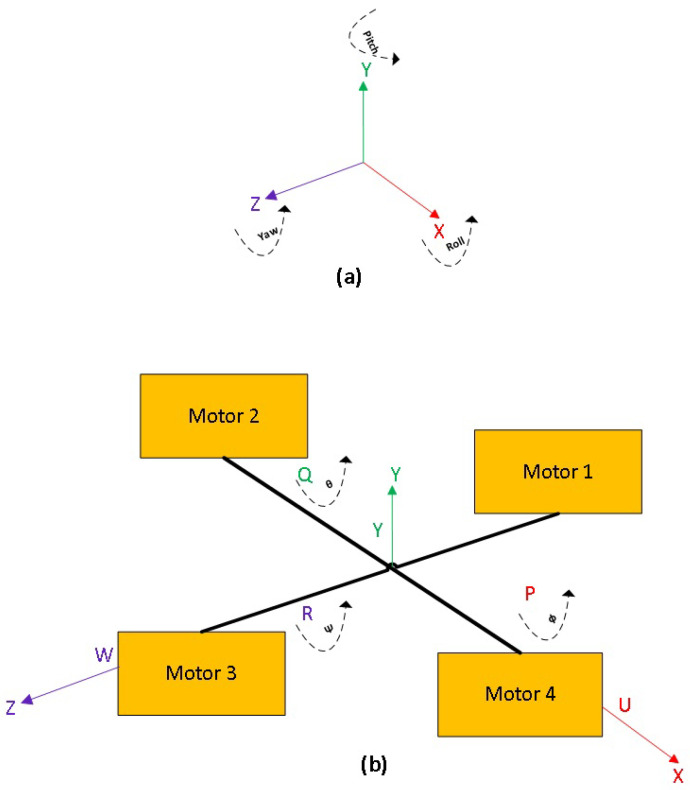
(**a**) Reference system of Quad-copter and (**b**) its movement with respect to the axes.

A mathematical model is a description of a system using mathematical concepts and language, and it plays its role in the operation of the device [63,64,65]. Similarly, the quad-copter’s aerodynamics is mathematically modelled with respect to two reference systems which are most important parameters to gain the stable flight and motion [41,42]. The first reference system is the inertial frame of reference which is related to the earth and the body of the quad-copter. The second frame of reference is related to the quad-copter’s frame itself, as to how how its translational and rotational motion can be controlled (portraying the frame orientation with respect to the origin of the quad-copter frame, Figure 4).

The position of the quad-copter during flight is given by the parameters Roll (ϕ), Pitch (θ) and Yaw (Ψ) angles, which define its rotation with respect to x-axis, y-axis and z-axis, respectively. The mathematical equation which defines the relationship between the quad-copter with respect to the earth is given by

(1) $$R=\left[\begin{array}{c} C_{\Psi }C_{\theta }\;C_{\Psi }S_{\theta }S_{\phi }-S_{\Psi }C_{\phi }\;C_{\Psi }S_{\theta }C_{\phi }-S_{\Psi }C_{\phi }\\ S_{\Psi }C_{\theta }\;S_{\Psi }S_{\theta }S_{\phi }-C_{\Psi }C_{\phi }\;S_{\Psi }S_{\theta }C_{\phi }-C_{\Psi }S_{\phi }\\ -S_{\theta }C_{\theta }S_{\phi }\;\;\;C_{\theta }C_{\phi } \end{array}\right],$$

where $S_{\phi }$ & $C_{\phi }$ are notations of sin(x) and cos(x), respectively.

The thrust is the force or pull by which the quad-copter propellers move the air downwards for gaining upward force. Mathematically,

(2) $$T=k\cdot \omega^{2}.$$

The rotational torque of the quad-copter is proportional to the square of the angular velocity of quad-copter motors which is given as

(3) $$\tau_{\psi }={\left(-1\right)}^{i+1}b\cdot \omega_{i}^{2},$$

where motor *i* in the above equation is positive if the rotation of propeller is clockwise and negative otherwise. The torque is defined as the cross product of applied force and moment arm from the pivot point. Therefore, if *L* defines the length between a propeller and the copter’s mid-point, the total torque of the body of the frame of the quad-copter is given by [78]

(4) $$\tau_{B}=\left[\begin{array}{c} L.k.(-\omega_{1}^{2}+\omega_{3}^{2})\\ L.k.(-\omega_{2}^{2}+\omega_{4}^{2})\\ b.(-\omega_{1}^{2}+\omega_{2}^{2}-\omega_{3}^{2}+\omega_{4}^{2}) \end{array}\right].$$

### 4.3. Brushless Dc Motor Model

**Figure 5:**
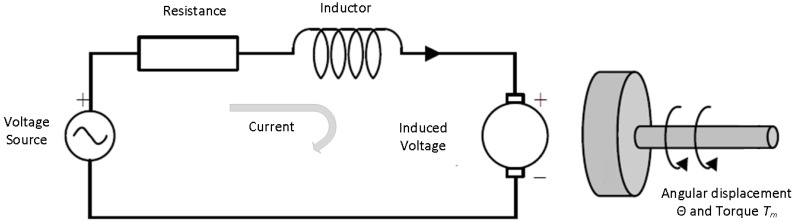
Mathematical Model of BLDC Motor.

The mathematical modelling of quad-copter motors is implemented by defining the mathematical model [79], which is shown in Figure 5.

Now, to find the transfer function of the model, we use armature voltage V(t) and angular displacement Θ, to define the angular velocity as

(5) $$\omega \left(t\right)=\frac{d\Theta }{dt}.$$

The quad-copter brushless motor mathematical model consist of both electrical and mechanical parts. The equation for the electrical phenomenon is as follows:

(6) $$-e\left(s\right)+R_{a}i_{a}+L_{a}\frac{di}{dt}+V_{b}=0,$$

where $V_{b}$ is the Back Electromotive Force which is given by

(7) $$V_{b}=K_{b}\omega ,$$

where $K_{b}$ is the motor constant.

**Mechanical Characteristics**

According to law of conversation of energy, the total sum of torques of motor must be equal to zero. Therefore,

(8) $$T_{e}-T_{\omega_{{}^{\prime }}}-T_{\omega }-T_{L}=0,$$

where $T_{e}$ is the electromagnetic torque, $T_{\omega_{{}^{\prime }}}$ is the torque due to rotational acceleration of the motor, $T_{\omega }$ is the torque generated due to velocity of the motor, and $T_{L}$ is the torque due to mechanical load across motor. $T_{e}$ is directly proportional to the armature current $i_{a}$ and it can be written as

(9) $$T_{e}=K_{t}i_{a},$$

where $K_{t}$ is torque constant and it depends on flux density of the stator magnets. $T_{\omega_{{}^{\prime }}}$ can be written as

(10) $$T_{\omega^{{}^{\prime }}}=J\frac{d\omega_{a}}{dt},$$

where *J* is the inertia of constant.

## 5. Experimental Results

This section presents the various results and outputs based on real-time processing using a controller. As part of our project, which involves obstacle avoiding, and stable autonomous flight, we wanted to determine whether it was possible to receive correct output from the ultrasonic and other peripheral devices and sensor by given input. Thus, for this purpose, a test bench is designed for simulation. The mathematical simulation was carried out in Matlab (student version, complete details are available on https://www.mathworks.com/help/pdf_doc/matlab/rn.pdf; accessed on 21 December 2021)). For the purpose of our work, no toolboxes were used, as our system did not find any similarity with them. These were conducted in consultation with the technical team, and the simulations were performed at least thrice before proceeding with their interpretations. Test results and transformed useful data after analysis are shown in this section. The results and data obtained from GPS after applying algorithm are also described here, in accordance with Table 2.

### 5.1. GPS Simulations

The GPS module used to find the current and desired locations of the quad-copter are presented here. As the purpose of GPS is to find the latitude and longitude, these two parameters are used to track the location of the quad-copter. The specifications of GPS used for autonomous quad-copter are as follows [80].

The Algorithm 1 used for the motion of quad-copter using GPS is *divide and conquer* [81,82,83,84], which is described below.
**Algorithm 1**: Divide and conquer1. Sort points along the *x*-coordinate.2. Split the set of points into two equal-sized subsets by a vertical line $x=x_{mid}$.3. Solve the problem recursively in the left and right subsets. This will give the left-side and right-side minimal distances $d_{Lmin}$ and $d_{Rmin}$, respectively.4. Find the minimal distance $d_{LRmin}$ among the pair of points in which one point lies to the left of the dividing vertical and the second point lies to the right.5. The final answer is the minimum among $d_{Lmin}$, $d_{Rmin}$, and $d_{LRmin}$.

**Figure 6:**
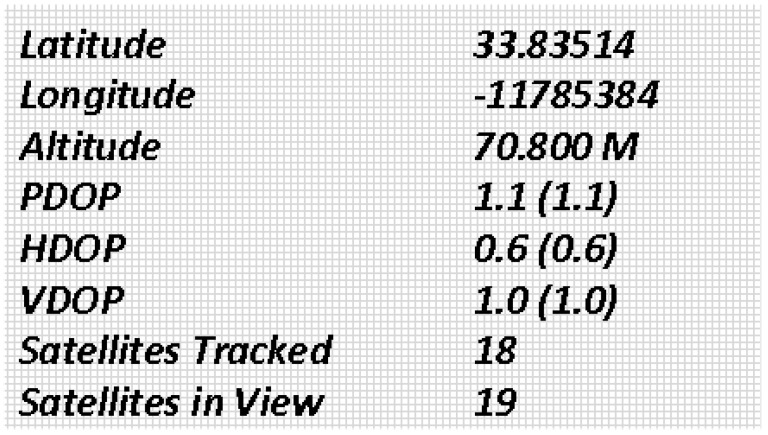
GPS Module output results.

The output results of the GPS module are shown in Figure 6.

### 5.2. Proportional (P), Integral (I) and Derivative (D) Controller

**Figure 7:**
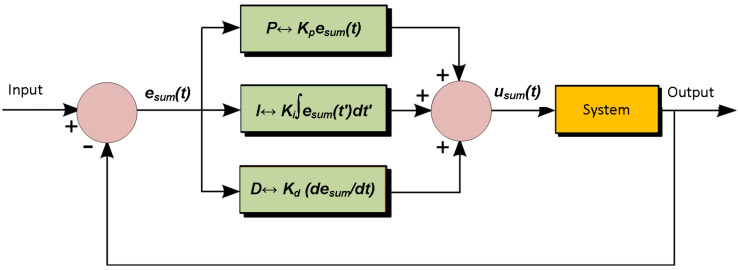
Working principle of a PID controller with feedback mechanism, indicating various components.

A Proportional (P), Integral (I) and Derivative (D) controller is the combination of three different types of controlling devices which are based on the control algorithm, hereby referred to as a PID controller. The mathematical modelling of quad-copter motors is performed by defining the mathematical model which is shown in Figure 7. A complete control mechanism becomes [31,32]

(11) $$\begin{array}{cc} u_{sum}\left(t\right)= & K_{p}e_{sum}\left(t\right)+K_{i}\int_{0}^{t}e_{sum}\left(t^{\prime }\right)dt^{\prime }+K_{d}\frac{de_{sum}\left(t\right)}{dt},\\ \; & K_{p}>0,K_{i}>0,K_{d}>0, \end{array}$$

where $K_{p},K_{i}$ and $K_{d}$ are coefficients for the corresponding Proportional (P), Integral (I) and Derivative (D) terms, respectively. Ideally, the quad-copter should directly attain a specific height, after a specific time, without causing any delay in the flight. For this purpose, we take this ideal flight to be 1 arbitrary units (a.u.), after the passage of exactly 1 s. This helps us in obtaining a comparison of the various parameters of the device with this standard (reference) graphical values and the corresponding results.

#### 5.2.1. Proportional Controller

**Figure 8:**
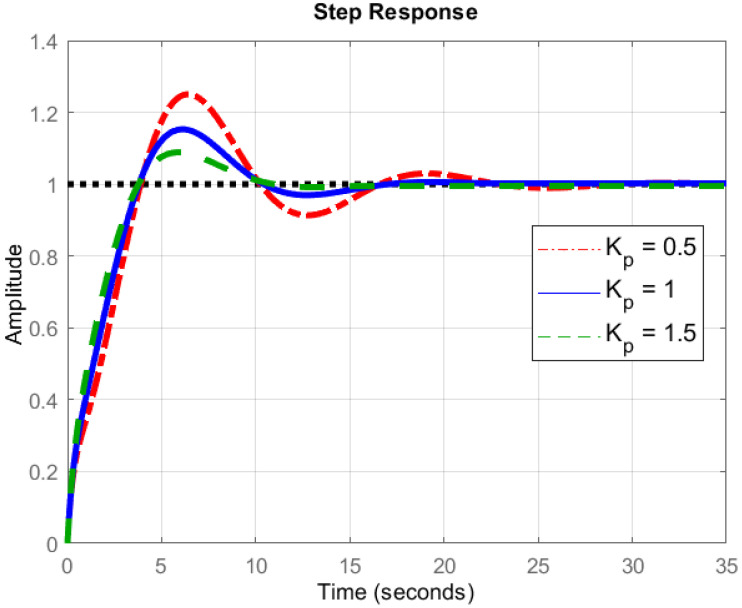
Principle of proportional controller.

It is very important to supply the correct amount of current to the quad-copter, as an error could lead to malfunctioning of the motor. For this purpose, the proportional controller is used which causes the motor current of quad-copter set in proportion to the error, as shown in Equation (11) and Figure 7, and the resulting plot is shown in Figure 8. However, the proportional controller fails if the arms of the quad-copter have to pull different weights during flight due to different environment disturbances. This is exactly where the derivative and integral controllers play their part.

#### 5.2.2. Integral Controller

**Figure 9:**
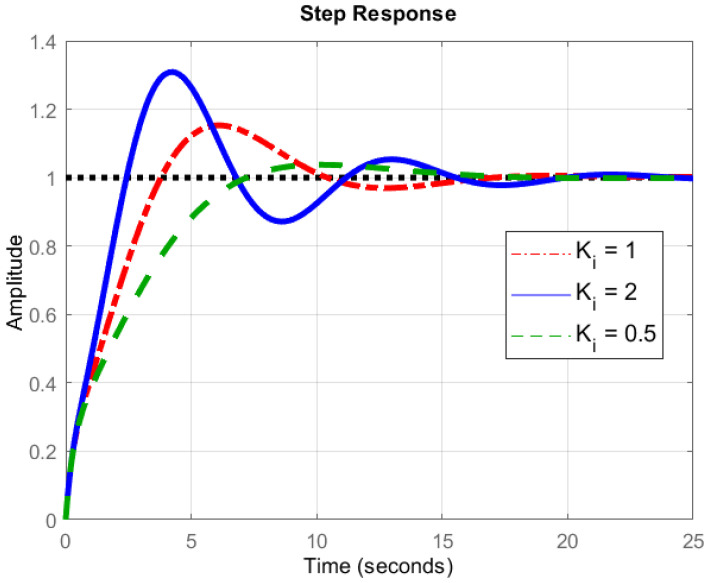
Principle of Integral Controller.

In this controller, the product of integral gain and error of the signal is added to the previous state of the model (Equation (11)), as indicated in Figure 7, with the resulting situation in Figure 9. The integral controller helps to decrease the rise time, thereby minimizing steady-state error. The integral term is proportional to both the magnitude of the error signal and the duration of the error in PID controller response. The different values of integral controller response are shown in Figure 9. A comparison among these values indicates the role of the integral controller in the flight of the device. For instance, when compared with the standard (reference) value, we see that the value of 0.5 shows the best closeness to the reference value, and the value of 2 shows the most different pattern. However, it must be stated that the time obtained by the quad-copter to attain a stable height is a bit more in case of the former value (0.5) as compared to the latter one (2). This could be justified by examining Equation (11), where a value of 2 will take a longer time as compared to 0.5, as the integration term will take time. This, in turn, will fluctuate the values, until a stable height is finally reached. This is exactly what is found in Figure 9.

#### 5.2.3. Derivative Controller

**Figure 10:**
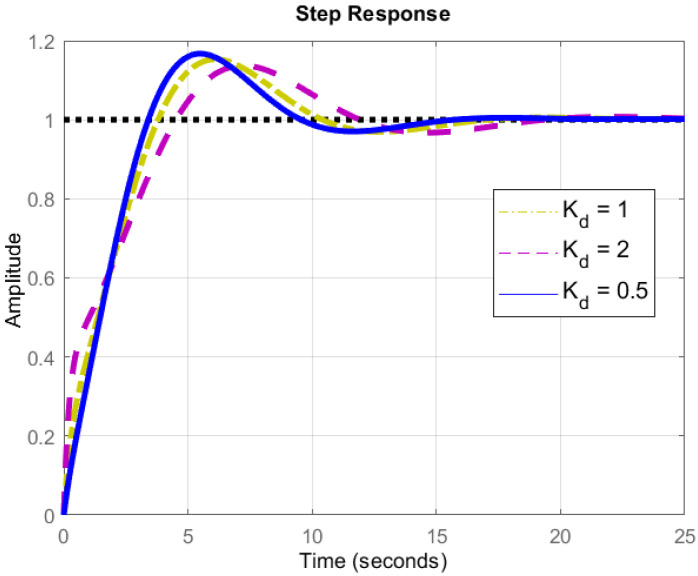
Principle of Derivative Controller.

The derivative controller is an important part of the PID controller which reacts only when there is a change in error, as per Equation (11). It protects the auto controller from surpassing its limit by generating friction. The derivative of the error signal is calculated by finding the slope of the error signal with time and product of this rate of change with the derivative gain Kd. The response of the derivative controller is shown in Figure 10. An examination of this figure can be correlated with the mathematical interpretation of Equation (11). A value of 2 will take more time to integrate (summing the component values), and this results in an relative fluctuating curve. This will continue until and unless a stable amplitude has been attained, which is desirable for the quad-copter, and mandatory for the transportation of medical items. Therefore, this fact must be kept into account during the operation of the quad-copter, likewise discussed in the former section in detail.

### 5.3. Trajectory of the Quad-Copter

In order to maintain a smooth test of the vehicle, some technical aspects have to be brought into focus:

1. With the help of the vehicle’s camera, a device can be detected by the vehicle using RF-ID tag on the object. This means that the object can be picked up from the hospital’s store where it is located in a certain shelf, and transported to the patient in need;
2. To test the efficiency of the drone’s activity, we place the central location of the drone within 300 m of the hospital’s store (at furthest);
3. The medicines which have to be transported from the hospital to the patients come in various forms, and are sensitive to environmental variations. At this stage, the vehicle is used to transport only solid medicines and devices, as per recommendations of the physicians of the concerned hospital;
4. Afterwards, the positions of the patients were set at random distances (displacements) from the hospital’s store, with the furthest one being at 1.5 km;
5. The maximum weight which the vehicle can lift is 1.5 kg. The maximum speed without any load is 25 km/h, and that with the maximum weight aboard is 21.5 km/h;
6. To carry out the experiments, specific permission was obtained from the local authority, as well as the hospital administration on weekends, as the work was not possible otherwise [43];
7. The weather conditions need to be taken into consideration beforehand. Each measurement was taken on a sunny day, with maximum wind speed of 8 km/h, atmospheric pressure under 1025 hPa, precipitation under 0.5 cm, humidity under under 65%, and visibility under 10.35 km.

**Figure 11:**
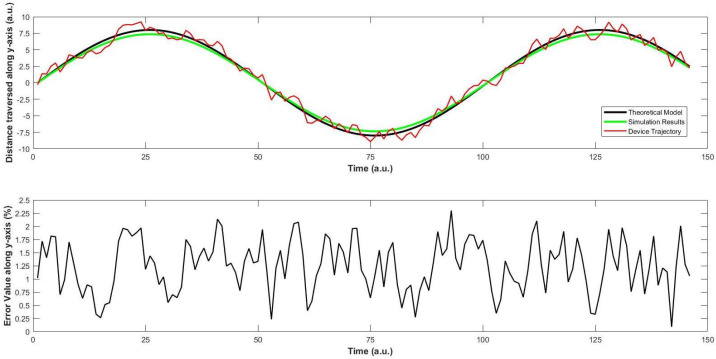
First trajectory scenario: Investigation of change in values of y. (**top**) shows a comparison between the ideal, simulation and actual (trajectory) values during the movement of the quad-copter along the y-axis, and (**bottom**) shows the average error between the simulated results and the actual (trajectory) values, which is very small.

In order to check the movement of the quad-copter from source to target destination, we resort to checking its trajectory along various directions. For the sake of convenience, the direction of motion towards the sky (vertical/upward) is nominated as the y-axis, the direction of motion towards right or left is nominated as the z-axis, and the direction towards the destination (horizontal) is nominated as the x-axis. This is conducted in complete accordance with Figure 4. First, we keep the values of x and z axes as constant, and vary the value of y. The motion of the quad-copter was recorded with the help of a video camera. The machine was flown five times along this path, and the average value was taken for each measurement. This value was compared with the simulated results, and the average error between these two values were calculated. These are shown in the respective plot in Figure 11.

**Figure 12:**
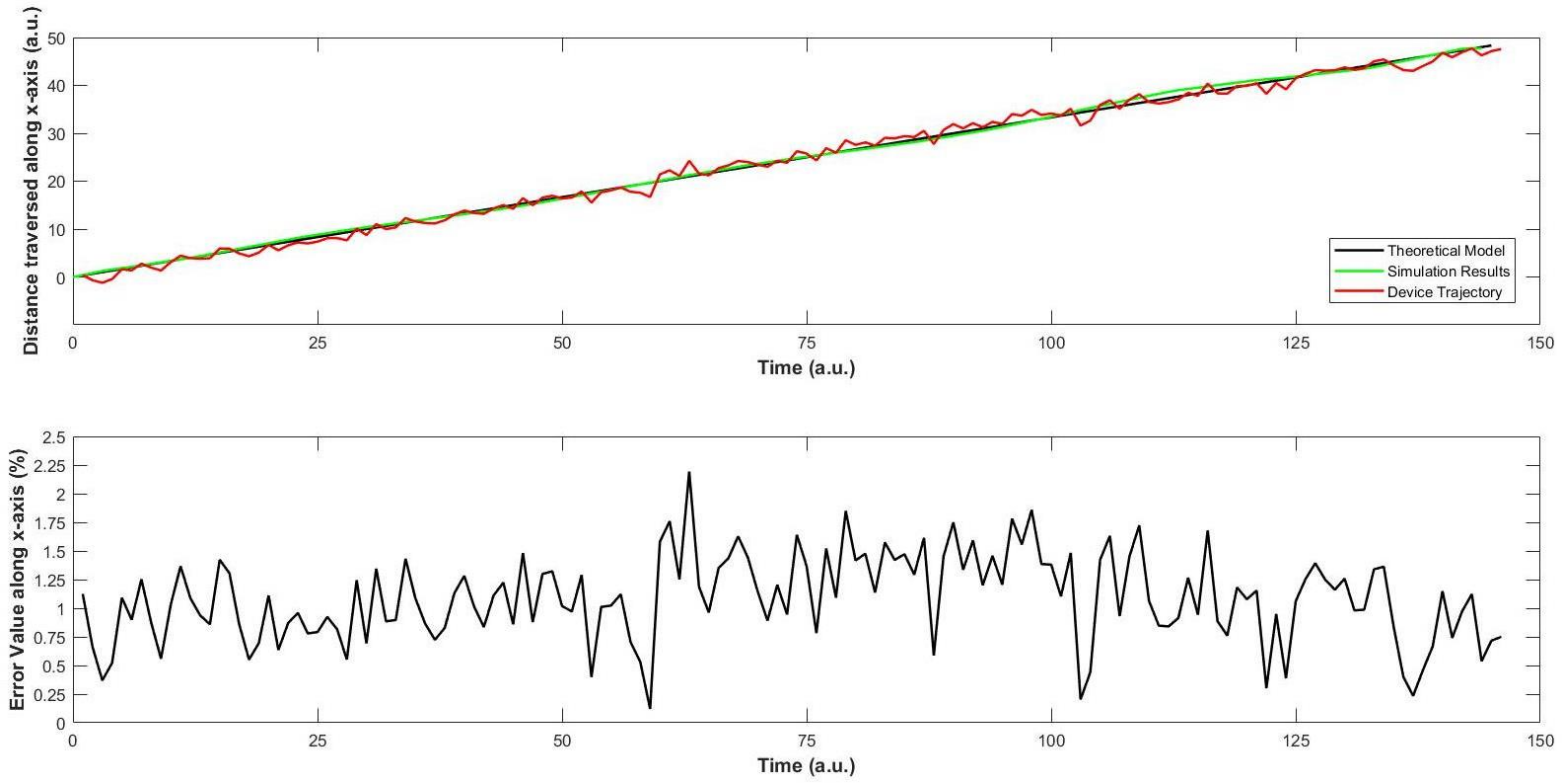
Second trajectory scenario: Investigation of change in values of x. (**top**) shows a comparison between the ideal, simulation and actual (trajectory) values during the movement of the quad-copter along the x-axis, and (**bottom**) shows the average error between the simulated results and the actual (trajectory) values, which is very small.

**Figure 13:**
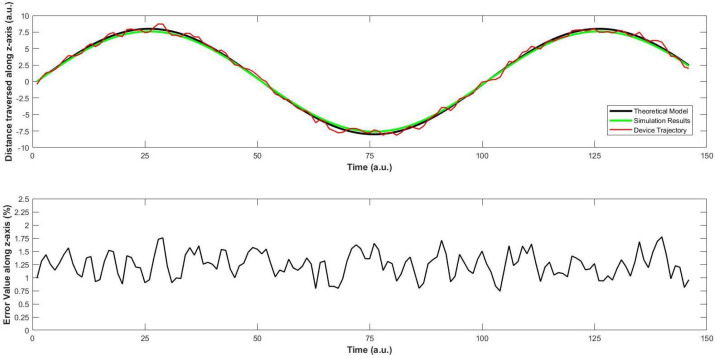
Second trajectory scenario: Investigation of change in values of z. (**top**) shows a comparison between the ideal, simulation and actual (trajectory) values during the movement of the quad-copter along the z-axis, and (**bottom**) shows the average error between the simulated results and the actual (trajectory) values, which is very small.

Next, we perform the same test along x-axis, while keeping constant the values along the other two axes. The actual values were compared with the simulation results, and they are shown in Figure 12. Similarly, the case is analysed along the z-axis in Figure 13.

*Observations*

1. In Figure 11, simulation results show resemblance with the theoretical ones, as well as the trajectory of the quad-copter. As soon as the device takes off, we see that the error between the simulation and trajectory is less than 1.75%, which increases to a maximum of 2.25% at two occasions, efficiently comparable to the nearest results [21]. First, it is the occasion when the device has consumed about one-third of the travel time. This might be due to the sudden increase in the wind speed at that moment. A similar moment is observed when the device is about to reach the destination. On average, the error value along the ordinate is 1.17%, which is acceptable for a quad-copter in similar designs [15,21].
2. Regarding Figure 13, a similar trend is observed for the motion of the device along z-axis. The fluctuations in the trajectory are slightly more than those along the y-axis. This is mainly because of two reasons. First, the device is equipped with a sensor that checks its motion along y-axis, but not along the z-axis. After consultation with the local vendors, we could not find a particular solution at that moment. Second, when the air moves along any direction, it has an effect on the motion of the device. This matter was discussed with two pilots of helicopters, who agreed with our stance that the weight of this machine is much smaller than that of a normal helicopter, and this can have an effect on the motion along the z-axis. In addition, they said that this would supposedly not affect any objects loaded on the machine, unless they cross the weight limit of our quad-copter. The average error is found to be 1.28%, which is comparable to recent works on quad-copters with different applications [6,22].
3. Afterwards, the motion of the quad-copter along the x-axis is recorded and compared with the simulation results in Figure 12. When the device travels about half of its distance, some fluctuations are seen in this trajectory which can be interpreted as follows. The quad-copter leaves the store inside the hospital and flies over the ground along its way to the destination which is about half-way. On account of the open area, the air speed is slightly higher as there is less congestion. This again acts as a slight resistance for the device, on its way. Therefore, the device experiences some fluctuations at this point. The average error in the value is 1.04%, which is slightly less than for the y- and z-axes, and no correlated results could be found at this level [9,11].
4. As per the trajectory profile of the device, it is important to note the stability during its movement. At this moment, it is observed that the overall results are within tolerable limits that are the primary focus of the biomedical application. For BCI to further accelerate its progress, the size of the device is important, as discussed in [8,9]. This becomes crucial as the medication becomes sensitive, which is not found in [15]. Although it is successful in imaging issues, the approach in [19] needs to be verified in different weather conditions, streamlining the identical repercussions. This becomes more interesting as there has been a focus on testing and implementation of BCI in virtual environments [21], and much remains to be done for the practical scenario, as we have approached here, with positive prospects in the future. This requires a deep investigation of the device in various dimensions on a continuous basis with BCI, an approach that has been attempted for the first time hitherto.
5. In this manner, our focus in this work was to implement the controller for better control of the quadcopter that can be used to implement the real brain signals as in the literature [11,15,16,17,22]. The target is to implement it in a biomedical sensor for which various technical aspects have been investigated. The characteristics of the controller were discussed in detail for smooth functioning with the real brain signals that can aid in the prospective design of the said scheme in the future.

## 6. Conclusions

The proposed framework provided better results in controlling air-vehicles with less error which can be engaged for brain–computer interfaces. In this manner, this research continues in light of recent developments in the areas of UAVs, as there is an urgent need to develop systems that are able to deliver logistics in a reliable and safe way. This becomes more important for the case of biomedical sensors that are designed to be mounted on the human body. In case of an emergency situation, the physician needs to take immediate steps, which might be life-saving in certain situations, and a direct connection between the physician and the patient might certainly not be possible on account of distance. The sensitivity of such a scenario raises a red flag if the locality of the patient is in a rural or distant area, with unspecified transportation options. This was the motivation behind the development of our biomedical sensor that is designed to monitor the health status of elderly patients. Considering the need to transport medical equipment from the hospital to the patient and vice versa, the current work tackles the development of a UAV in the form of a quad-copter. Several issues have been dealt with during the development of this device, after obtaining special permission to check the device in an urban environment. The location of the medical centre and the patient play an important role in the experimentation, for which the investigation was conducted appropriately. The maximum value of average error between simulation and trajectory was found to be at 1.368% in all cases, with a load carrying capacity of 2 kg, under persistent weather conditions that have been mentioned hitherto. This means that not only medication can be supplied, but also some equipment in this range of weight can be moved from the hospital to the affected person which can help in saving precious lives. Another novel aspect is the fact that the equipment which has been used for this purpose is not very expensive, aiding in the physical implementation of the biomedical sensor in terms of its economic parameters. Care has been taken to analyse the device in specific conditions, maintaining the sanctity, precision and accuracy of the work, justified by the physicians of the hospital.

There are numerous ideas that can be explored to extend this work. One of the future work directions of this project is to enhance quad-copter trajectory by implementing machine learning-based scheme to remember the path of trajectory of flight, in order to maximize the logistics of medication. At this time, the device can lift 2 kg, and reviews are being performed extend this range. The experimentation has been satisfactorily done in the prescribed weather conditions (daylight, partly cloudy and windy). Since the weather in the surrounding of the experimental range is like this, we are negotiating with the technical team to extend it to harsher conditions such as rain and snow in the future, and this might require certain permissions from the aviation authority in those areas. We hope that once the results become available, they would lead to supply of medication to the far-off areas requiring a much smaller amount of time, thereby saving precious resources. For this, we are planning to map the vicinity of the medical centre where the UAV is expected to manoeuvre whenever required. Once this is done, the device might be able to fly to the desired location by using the least time and resources, which is one of the main targets of automated vehicles for the future.

## Figures and Tables

**Table 1:** List of symbols.

VC_i_/VC1	Variable Coupler corresponding to the inner cavity
VC_o_/VC2	Variable Coupler corresponding to the outer cavity
SOA	Semiconductor Optical Amplifier
OSA	Optical Spectrum Analyzer
M_i_/M1	Mode corresponding to the inner cavity
M_o_/M2	Mode corresponding to the outer cavity
UAV	Unmanned Air Vehicle
ESC	Electronic Speed Controller
BLDC	Brushless Direct Current
BEC	Battery Elimination Circuit
Li-Po	Lithium Polymer
ϕ	Roll
θ	Pitch
Ψ	Yaw
$V_{b}$	Back Electromotive Force
$T_{e}$	Electromagnetic Torque
$T_{\omega_{{}^{\prime }}}$	Torque due to rotational acceleration of motor
$T_{\omega }$	Torque generated due to velocity of the motor
$T_{L}$	Torque due to mechanical load across motor
$K_{t}$	Torque constant
*J*	Inertia of constant
$K_{p}$	Coefficient for Proportional term
$K_{i}$	Coefficient for Integral term
$K_{d}$	Coefficient for Derivative term

**Table 2:** Description of main parameters.

No.	Parameter	Description
1	Satellites	22 tracking, 66 searching
2	Patch Antenna Size	15 mm × 15 mm × 4 mm
3	Update rate	1 to 10 Hz
4	Position Accuracy	1.8 m
5	Velocity Accuracy	0.1 m/s
6	Warm/cold start	34 s
7	Acquisition sensitivity	−145 dBm
8	Tracking sensitivity	−165 dBm
9	Maximum Velocity	515 m/s
10	Input Voltage range	3.0–5.5 V DC
11	Current drawn during navigation	25 mA tracking, 20 mA
12	Output	NMEA 0183, 9600 baud default
13	Feature	Multi-path detection and compensation

## Data Availability

Not applicable.
